# *EXO - Beyond the Cell,* a journal about how cells interact with their environment

**DOI:** 10.70401/EXO.2026.0001

**Published:** 2026-01-13

**Authors:** Brent R. Stockwell

**Affiliations:** 1 Department of Biological Sciences, Columbia University, New York, NY 10027, USA.; 2 Department of Chemistry, Columbia University, New York, NY 10027, USA.; 3 Irving Institute for Cancer Dynamics, Data Science Institute, Columbia University, New York, NY 10027, USA.; 4 Department of Pathology and Cell Biology, Herbert Irving Comprehensive Cancer Center, Columbia University Digestive and Liver Disease Research Center, Vagelos College of Physicians and Surgeons, Columbia University Irving Medical Center, New York, NY 10032, USA.

I’m pleased to announce the launch of *EXO - Beyond the Cell*, a new journal focused on how cells interact with their environment. In recent years, there has been an explosion of methods, data, and analyses of spatial data, revealing that the geography of cells is critical in tissue contexts^[1]^. Studying cells in isolation misses this critical aspect of how cells engage with their native environment; interactions between cells in tissues have a profound impact on cell and organ biology, and important implications for medicine^[2]^. Tissue interactions are critical in all areas of biology, from neuroscience^[3]^ to immunology^[4]^ to cancer biology^[5]^ and far beyond.

As in most scientific revolutions, this new field of spatially-informed biology has required the development of new methods and analytical frameworks^[6]^. These include systematic and high-throughput global methods of data collection, including spatial transcriptomics, proteomics and metabolomics, as well as computational methods for multi-omic integration, 3D reconstructions, and defining spatial gradients, to name a few examples. With these new tools, we can now identify not just the different cell populations present in tissues, but also how they interact and how those interactions create different cell subtypes and states that drive normal physiology and pathophysiology. Indeed, the next century is likely to be one that is rich in spatial data, providing opportunities for deeper understanding, as well as improved diagnostic tools and therapeutic interventions.

*EXO* aims to be the premier journal for publishing rigorous and impactful studies, reviews, and commentaries on how cells interact with their environment. We are excited to receive papers on new technologies, tools, and applications in this frontier. I’m thankful to the truly outstanding members of our Editorial Board who have agreed to guide the journal and help us realize this mission. If you peruse their profiles on the website, you will find that we have exceptional scientists who are leaders in all aspects of this emerging field, and hence, manuscripts will be thoughtfully and carefully assessed. Regarding my own journey into spatial biology, my lab at Columbia University began exploring spatial tools for metabolism, including mass spectrometry methods, nearly a decade ago, stemming from our interest in how lipids and metabolites influence susceptibility to ferroptosis, a regulated form of cell death we identified in 2003^[7]^, and then thoroughly defined in a series of papers from then through 2012^[8–11]^, using the tools of chemical biology^[12,13]^. We indeed have seen over many years that spatial data are critical for understanding in vivo metabolism^[14–16]^, pharmacology^[17,18]^, chemical biology data^[19–21]^, and more generally, cell biology^[22–27]^. Hence, my excitement about leading a journal dedicated to this field.

Let me turn to the journal itself and tell you some of the innovative features we are implementing. The journal is open access and hence there are no subscription fees; additionally, there won’t be any article processing charges (APCs) until 2030. We have also launched a *Founding Contributor Program* to reward authors who publish in the journal in the first year. The first author or corresponding author of each review or primary article published before Nov 30th, 2026 will receive an APC waiver for use after 2030, when modest APCs are introduced. The journal will also provide a *Founding Contributor Certificate* and membership in our *Founding Contributor Honor Wall,* as well as eligibility for the *Founding Author Award* that we will announce after this period. We hope that these incentives will encourage the community to submit their best ideas and work to *EXO* so that we all benefit from a truly exceptional place to publish our findings and ideas.

I plan to be involved in each submission, ensuring that the journal provides authors with transparent, constructive, and effective feedback. We want to limit reviewer feedback to a handful of critical questions about whether the data support the conclusions, so that we have rigorous and exceptional papers, but not unnecessarily requested experiments that drain time and resources. We want reviewers and authors to enjoy the experience of publishing in *EXO.* We also want to showcase *EXO* papers so that students and postdocs gain the benefit of visibility to help them in their next career step, becoming leaders in the spatial biology community upon publishing in EXO. I want to hear from our prospective authors – have we done a good job, and if not, how can we do better?

With many consortia, including HuBMAP^[28]^, the Human Cell Atlas^[29]^, and the Human Tumor Atlas Network^[30]^ focused on mapping cells in their tissue contexts, we can see that spatial, single-cell biology is the next major frontier in biology and medicine. I look forward to watching this revolution unfold in the pages of EXO.

## References

[1] Liu L, Chen A, Li Y, Mulder J, Heyn H, Xu X. Spatiotemporal omics for biology and medicine. Cell. 2024;187(17):4488–4519. [DOI] 39178830 10.1016/j.cell.2024.07.040

[2] Skinnider MA, Courtine G, Bloch J, Squair JW. A clinical road map for single-cell omics. Cell. 2025;188(14):3633–3647. [DOI] 40645168 10.1016/j.cell.2025.06.009

[3] Nowakowski TJ, Nano PR, Matho KS, Chen X, Corrigan EK, Ding W, The new frontier in understanding human and mammalian brain development. Nature. 2025;647(8088):51–59. [DOI] 41193845 10.1038/s41586-025-09652-1 PMC13108688

[4] Kamiya M, Carter H, Espindola MS, Doyle TJ, Lee JS, Merriam LT, Immune mechanisms in fibrotic interstitial lung disease. Cell. 2024;187(14):3506–3530. [DOI] 38996486 10.1016/j.cell.2024.05.015 PMC11246539

[5] Seferbekova Z, Lomakin A, Yates LR, Gerstung M. Spatial biology of cancer evolution. Nat Rev Genet. 2023;24(5):295–313. [DOI] 36494509 10.1038/s41576-022-00553-x

[6] Rood JE, Hupalowska A, Regev A. Toward a foundation model of causal cell and tissue biology with a Perturbation Cell and Tissue Atlas. Cell. 2024;187(17):4520–4545. [DOI] 39178831 10.1016/j.cell.2024.07.035

[7] Dolma S, Lessnick SL, Hahn WC, Stockwell BR. Identification of genotype-selective antitumor agents using synthetic lethal chemical screening in engineered human tumor cells. Cancer Cell. 2003;3(3):285–296. 12676586 10.1016/s1535-6108(03)00050-3

[8] Dixon SJ, Lemberg KM, Lamprecht MR, Skouta R, Zaitsev EM, Gleason CE, Ferroptosis: an iron-dependent form of nonapoptotic cell death. Cell. 2012;149(5):1060–1072. [DOI] 22632970 10.1016/j.cell.2012.03.042 PMC3367386

[9] Yang WS, Stockwell BR. Synthetic lethal screening identifies compounds activating iron-dependent, nonapoptotic cell death in oncogenic-RAS-harboring cancer cells. Chem Biol. 2008;15(3):234–245. [DOI] 18355723 10.1016/j.chembiol.2008.02.010 PMC2683762

[10] Yagoda N, von Rechenberg M, Zaganjor E, Bauer AJ, Yang WS, Fridman DJ, RAS-RAF-MEK-dependent oxidative cell death involving voltage-dependent anion channels. Nature. 2007;447(7146):864–868. [DOI] 17568748 10.1038/nature05859 PMC3047570

[11] Stockwell BR. Ferroptosis turns 10: Emerging mechanisms, physiological functions, and therapeutic applications. Cell. 2022;185(14):2401–2421. [DOI] 35803244 10.1016/j.cell.2022.06.003 PMC9273022

[12] Stockwell BR. Chemical genetics: Ligand-based discovery of gene function. Nature Reviews Genetics. 2000;1(2):116–125. [DOI] 10.1038/35038557 PMC3171795 11253651

[13] Stockwell BR. Exploring biology with small organic molecules. Nature. 2004;432(7019):846–854. [DOI] 15602550 10.1038/nature03196 PMC3165172

[14] Wong Fok Lung T, Charytonowicz D, Beaumont KG, Shah SS, Sridhar SH, Gorrie CL, Klebsiella pneumoniae induces host metabolic stress that promotes tolerance to pulmonary infection. Cell Metab. 2022;34(5):761–774. [DOI] 35413274 10.1016/j.cmet.2022.03.009 PMC9081115

[15] Sugimoto A, Saito Y, Wang G, Sun Q, Yin C, Lee KH, Hepatic stellate cells control liver zonation, size and functions via R-spondin 3. Nature. 2025;640(8059):752–761. [DOI] 40074890 10.1038/s41586-025-08677-w PMC12003176

[16] Tian H, Rajbhandari P, Tarolli J, Decker AM, Neelakantan TV, Angerer T, Multimodal mass spectrometry imaging identifies cell-type-specific metabolic and lipidomic variation in the mammalian liver. Dev Cell. 2024;59:1–13. [DOI] 38359832 10.1016/j.devcel.2024.01.025 PMC11656446

[17] Linkermann A, Skouta R, Himmerkus N, Mulay SR, Dewitz C, De Zen F, Synchronized renal tubular cell death involves ferroptosis. Proc Natl Acad Sci U. A. 2014;111(47):16836–16841. [DOI] 10.1073/pnas.1415518111 PMC4250130 25385600

[18] Rajbhandari P, Neelakantan TV, Hosny N, Stockwell BR. Spatial pharmacology using mass spectrometry imaging. Trends Pharmacol Sci. 2024;45(1):67–80. [DOI] 38103980 10.1016/j.tips.2023.11.003 PMC10842749

[19] Root DE, Kelley BP, Stockwell BR. Detecting spatial patterns in biological array experiments. J Biomol Screen. 2003;8(4):393–398. [DOI] 14567791 10.1177/1087057103254282 PMC1262643

[20] Bailey SN, Sabatini DM, Stockwell BR. Microarrays of small molecules embedded in biodegradable polymers for use in mammalian cell-based screens. Proc Natl Acad Sci U. A. 2004;101(46):16144–16149. [DOI] 10.1073/pnas.0404425101 PMC528944 15534212

[21] Yozwiak CE, Hirschhorn T, Stockwell BR. Toward a Microparticle-Based System for Pooled Assays of Small Molecules in Cellular Contexts. ACS Chem Biol. 2018;13(3):761–771. [DOI] 29365249 10.1021/acschembio.8b00043 PMC5960808

[22] Agmon E, Solon J, Bassereau P, Stockwell BR. Modeling the effects of lipid peroxidation during ferroptosis on membrane properties. Sci Rep. 2018;8(1):5155. [DOI] 29581451 10.1038/s41598-018-23408-0 PMC5979948

[23] Gaschler MM, Hu F, Feng H, Linkermann A, Min W, Stockwell BR. Determination of the subcellular localization and mechanism of action of ferrostatins in suppressing ferroptosis. ACS Chem Biol. 2018;13(4):1013–1020. [DOI] 29512999 10.1021/acschembio.8b00199 PMC5960802

[24] Wu J, Minikes AM, Gao M, Bian H, Li Y, Stockwell BR, Intercellular interaction dictates cancer cell ferroptosis via NF2-YAP signalling. Nature. 2019;572(7769):402–406. [DOI] 31341276 10.1038/s41586-019-1426-6 PMC6697195

[25] Ye LF, Chaudhary KR, Zandkarimi F, Harken AD, Kinslow CJ, Upadhyayula PS, Radiation-induced lipid peroxidation triggers ferroptosis and synergizes with ferroptosis inducers. ACS Chem Biol. 2020;15(2):469–84. [DOI] 31899616 10.1021/acschembio.9b00939 PMC7180072

[26] Jin J, Schorpp K, Samaga D, Unger K, Hadian K, Stockwell BR. Machine learning classifies ferroptosis and apoptosis cell death modalities with TfR1 immunostaining. ACS Chem Biol. 2022;17(3):654–660. [DOI] 35230809 10.1021/acschembio.1c00953 PMC8938922

[27] Quardokus EM, Saunders DC, McDonough E, Hickey JW, Werlein C, Surrette C, Organ Mapping Antibody Panels: A community resource for standardized multiplexed tissue imaging. Nat Methods. 2023;20:1174–1178. [DOI] 37468619 10.1038/s41592-023-01846-7 PMC10406602

[28] Jain S, Pei L, Spraggins JM, Angelo M, Carson JP, Gehlenborg N, Advances and prospects for the Human BioMolecular Atlas Program (HuBMAP). Nat Cell Biol. 2023;25(8):1089–1100. [DOI] 37468756 10.1038/s41556-023-01194-w PMC10681365

[29] Rood JE, Maartens A, Hupalowska A, Teichmann SA, Regev A. Impact of the Human Cell Atlas on medicine. Nat Med. 2022;28(12):2486–2496. [DOI] 36482102 10.1038/s41591-022-02104-7

[30] Rozenblatt-Rosen O, Regev A, Oberdoerffer P, Nawy T, Hupalowska A, Rood JE, The human tumor atlas network: Charting tumor transitions across space and time at single-cell resolution. Cell. 2020;181(2):236–249. [DOI] 32302568 10.1016/j.cell.2020.03.053 PMC7376497

